# C2729T mutation associated with HBV mother-to-child transmission reduces HBV production via suppressing LHBs expression

**DOI:** 10.1080/21505594.2023.2189676

**Published:** 2023-03-15

**Authors:** Minmin Liu, Yarong Song, Yi Li, Xingwen Yang, Hui Zhuang, Jie Li, Jie Wang

**Affiliations:** a Department of Microbiology & Infectious Disease Center, School of Basic Medical Sciences, Peking University Health Science Center, Beijing, China; b NHC Key Laboratory of Medical Immunology, Peking University, Beijing, China

**Keywords:** Hepatitis B virus, mother-to-child transmission, preS1 promoter, large hepatitis B surface antigen, hepatocyte nuclear factor 1

## Abstract

Mother-to-child transmission (MTCT) is still the main route of hepatitis B virus (HBV) infection. However, the virological factors affecting HBV MTCT have not been fully elucidated. In this study, based on a prospective cohort of mother-infant pairs with positive maternal hepatitis B surface antigen (HBsAg), we found that the average nucleotide mutation rate of HBV preS1 promoter (SPI) region in the immunoprophylaxis success group was significantly higher than that in the immunoprophylaxis failure group. Among the nucleotide mutations of the HBV SPI region, the C2729T mutation had the highest frequency. Next, we found that the C2729T mutation promoted HBsAg release but reduced HBV production by suppressing the expression of large hepatitis B surface antigen (LHBs), and overexpressing LHBs could rescue this phenomenon. Based on the fact that the C2729T mutation could alter the binding site of hepatocyte nuclear factor 1 (HNF1) in the HBV SPI region, we uncovered that such an alteration could downregulate the transcriptional activity of SPI by attenuating the binding ability of HNF1 and HBV SPI region. This study suggests that HBV C2729T mutation may contribute to the immunoprophylaxis success of HBV MTCT by reducing HBV production, which supplements the virological factors affecting HBV MTCT.

## Introduction

According to the estimation of World Health Organization (WHO), there were 296 million people living with chronic hepatitis B virus (HBV) infection, and an estimated 820,000 cases died of HBV infection-related end-stage liver diseases including liver cirrhosis and hepatocellular carcinoma (HCC) in 2019 [1]. World Health Assembly (WHA) has proposed a global health sector strategy, which aims to reduce new HBV infections by 95% and viral hepatitis-related mortality by 65% by 2030 [2]. At present, there are still 1.5 million new cases of HBV infection every year, and mother-to-child transmission (MTCT) plays a great role in the transmission of HBV and leads to 50% of new HBV infections worldwide [3]. Although the full immunization of the hepatitis B vaccine (HepB) and hepatitis B immunoglobulin (HBIG) has significantly reduced the incidence of HBV MTCT in infants born to hepatitis B surface antigen (HBsAg)-positive mothers, 8–30% of mothers with high viral load may still transmit the virus to their children, resulting in immunoprophylaxis failure [4]. Therefore, the prevention of HBV MTCT should be a high priority in the process of eliminating hepatitis B.

HBV is a DNA virus with double-stranded relaxed circular DNA (rcDNA), which carries an approximately 3.2 kb long HBV genome. There are five HBV RNAs, including 3.5 kb pregenomic RNA (pgRNA) encoding HBV DNA polymerase and core protein (HBc), 3.5 kb preC mRNA encoding e antigen (HBeAg), 2.4 kb HBV RNA encoding large HBsAg (LHBs), 2.1 kb HBV RNA encoding middle (MHBs) and small HBsAg (SHBs), and 0.7 kb HBV RNA encoding HBx protein. The expression of HBsAg is regulated by two promoters of the *S* gene, including the preS1 promoter (SPI) regulating LHBs expression and the preS2 promoter (SPII) regulating MHBs and SHBs expressions. LHBs plays an important role in HBV replication, infection, and secretion [5,6]. The preS1 domain of LHBs is a key determinant for the binding of sodium taurocholate-cotransporting polypeptide (NTCP), which specifically mediates HBV entry into hepatocytes [7]. Meanwhile, an inappropriately high or low LHBs/SHBs ratio can lead to the inhibition of virion secretion, and overexpressing LHBs can also inhibit HBsAg release [6,8,9].

In terms of viral factors, HBV genome mutations have been proven to be associated with HBV MTCT [4,10,11]. As the main target of neutralizing B cell response, the mutations of HBV “α” determinant region may lead to immune escape by affecting the recognition of antibody to HBsAg (anti-HBs) to HBsAg, and thus may contribute to HBV MTCT [12]. However, we and other groups found that the mutations of “α” determinant region did not significantly affect the immunoprophylaxis effect of HBV MTCT [13,14]. Therefore, it is still unclear whether HBsAg mutations are responsible for immunoprophylaxis failure of HBV MTCT. Especially for the two promoter regions of the *S* gene, there is no relevant report on the impact of the mutations in the two promoter regions on the immunoprophylaxis effect of HBV MTCT.

In this study, based on a prospective cohort of mother-infant pairs with positive maternal HBsAg, we investigated the relationships between mutations in the promoter and coding region of the *S* gene and the immunoprophylaxis effect of HBV MTCT, and found that there were significant differences in SPI mutations between the immunoprophylaxis success and failure groups, including C2729T and G2765A mutations. It has been reported that G2765A mutation can significantly reduce HBV production by inhibiting the expression of LHBs [15], which may provide ideas for the mechanism of SPI mutations potentially affecting the immunoprophylaxis effect of HBV MTCT. Therefore, to clarify the effect of SPI mutations on HBV MTCT, we further explored the potential mechanism of C2729T mutation on HBV production in this study.

## Materials and methods

### Subjects

HBsAg-positive pregnant women were recruited in Jiangsu, Henan, and Jilin Provinces from 2009 to 2014. All infants received three doses of 10 μg or 20 μg recombinant yeast-derived HepB at birth (within 12 h), 1, and 6 months, combined with one dose of HBIG, and returned for post-vaccination serologic testing (PVST) at 7 months of age. According to the results of PVST, immunoprophylaxis failure was defined as positive HBsAg and anti-HBs levels<10 mIU/mL, and immunoprophylaxis success was defined as negative HBsAg and anti-HBs levels≥10 mIU/mL. Totally, 17 infants born to HBeAg-positive mothers with high viral loads (>7 log_10_ IU/mL) were immunoprophylaxis failure after the full course of vaccination, and were infected with genotype C2 HBV. Under the condition of strictly matching maternal HBsAg level, HBeAg status, viral load, HBV genotype, and ALT level with the immunoprophylaxis failure group, 17 eligible mother-infant pairs were randomly selected into the immunoprophylaxis success group.

### HBV genome amplification, sequencing, and sequence analysis

HBV DNA in serum samples was extracted by QIAamp DNA blood mini kit (Qiagen, Hilden, Germany). The full-length HBV genomes were amplified by polymerase chain reaction (PCR), followed by direct sequencing. The PCR primers and thirteen sequencing primers were described in Supplementary Table S1 and Table S2. The average nucleotide mutation rate = the total mutation frequency of the analysed region/(the number of nucleotides in the region × the number of sequences in the group) [16].

### Plasmids

The mutant HBV plasmid (pBB4.5-HBV1.2×-C2729T) and HBV SPI luciferase reporter plasmid (pGL3-SPI-C2729T) were generated by site-directed mutagenesis method from the wild-type 1.2-fold HBV plasmid (pBB4.5-HBV1.2×-WT, genotype C) and wild-type HBV SPI luciferase reporter plasmid (pGL3-SPI), respectively. The pCDH-LHBs plasmid was constructed by inserting the HBV LHBs coding region amplified from pBB4.5-HBV1.2×-WT into the pCDH vector using pEASY-Uniseamless Cloning and Assembly Kit (TransGen Biotech, Beijing, China) after digestion with BamHI. The pcDNA3.1-HNF1-HA plasmid was constructed by inserting the hepatocyte nuclear factor 1 (HNF1) with HA tag sequence amplified from HepG2 into the pcDNA3.1 vector after digestion with BamHI and XbaI. The primers for plasmid construction were shown in Supplementary Table S3.

### Cell culture and transfection

HepG2 and Huh7 cells were maintained as described previously [17]. The cells were transfected using Lipofectamine 3000 transfection reagent (Invitrogen, Carlsbad, CA, USA) according to the manufacturer protocol.

### Detection of HBsAg and HBeAg

HBsAg (quantification range: 0.05–250 IU/ml) and HBeAg (quantification range: 0.1–200 PEI U/mL) in the supernatants were quantified by chemiluminescence immunoassay kits (Autobio diagnostics Co., Zhengzhou, Henan, China) according to the manufacturer’s protocol.

### Western blot

The proteins in the cell lysates were separated via 10% SDS-PAGE and electrophoretically transferred onto PVDF membranes. After being blocked with 5% skim milk, the membranes were incubated with the relevant primary antibodies, including horse anti-HBs (Abcam, Cambridge, MA, USA), mouse anti-preS1 (Fitzgerald, Acton, MA, USA), and rabbit anti-β-actin (MBL, Nagoya, Japan), at 4°C overnight. On the second day, after incubating with the relevant secondary antibodies, including the HRP-linked goat anti-rabbit IgG (Easybio, Beijing, China), HRP-linked goat anti-mouse IgG (Easybio, Beijing, China), and HRP-linked rabbit anti-horse IgG (Abcam, Cambridge, MA, USA), at room temperature for 1 h, the band signals on the membranes were visualized by a ChemiDoc XRS Imaging System (Bio-rad, Hercules, CA, USA).

### Northern blot

Northern blot was performed to detect HBV RNAs as described previously [17]. Briefly, the heat-denatured total RNA was separated by a 1.5% agarose gel, and then transferred onto Nylon membranes (Roche, Basel, Kanton Basel, Switzerland) by capillary transfer and fixed by ultraviolet cross-linking. Then, the membranes were hybridized with a DIG-labelled HBV DNA probe, followed by incubation with anti-DIG-AP Fab fragments (Roche, Basel, Kanton Basel, Switzerland). Finally, the bands on membranes were exposed to ChemiDoc XRS Imaging System (Bio-Rad, Hercules, CA, USA). The DNA probe was generated by PCR with the forward primer: 5’-CTAATCATCTCATGTTCA-3,’ reverse primer: 5’-GGACTGCGAATTTTGGCC-3,’ and digoxigenin-11-dUTP.

### Luciferase reporter assay

HepG2 and Huh7 cells were co-transfected with pGL3-SPI or pGL3-SPI-C2729T plasmid and pRL-TK plasmid carrying renilla luciferase. At 48 h post-transfection, Dual-Luciferase Assay Kit (Promega, Madison, WI, USA) was used to measure the relative luciferase activity of cell lysate according to the manufacturer’s protocol.

As an internal control plasmid, nano-luciferase expression plasmid (pCDH-Nluc) was co-transfected with other plasmids in this study. At 72 h post-transfection, the Nano-Glo luciferase assay kit (Promega, USA) was used to detect the luciferase activity.

### Chromatin immunoprecipitation (ChIP)-quantitative real-time PCR (qPCR)

HepG2 and Huh7 cells harvested in 10 cm dishes were used to perform the ChIP assay by a Simple ChIP Enzymatic Chromatin IP Kit (Cell Signaling Technology, Danvers, MA, USA). The cells were co-transfected with pBB4.5-HBV1.2×-WT or pBB4.5-HBV1.2×-C2729T plasmid and pcDNA3.1-HNF1-HA. At 48 h after transfection, the cells were crosslinked with 1% formaldehyde and then were lysed with SDS lysis buffer. Next, DNA fragments were generated by sonication and incubated with anti-HA-tag (MBL, Nagoya, Japan). After immunoprecipitation, the DNA was purified and then detected by qPCR using the forward primer: 5′-TCCTGAACATGCAGTTAATC-3′, and the reverse primer: 5′-GTTCCCAAGAATATGGTGAC-3′.

### Quantification of extracellular HBV DNA and intracellular HBV RNA

After digesting the cell culture supernatants with DNase I (Takara, Tokyo, Japan) for 1 h at 37°C, the extracellular HBV DNA was extracted by a QIAamp DNA Blood Mini Kit (Qiagen, Hilden, Germany) according to the manufacturer’s protocol. As our previous report [17], the intracellular HBV RNA was extracted and reverse transcribed to cDNA. HBV DNA and RNA levels were measured by qPCR with SYBR Green method. The primers for measuring HBV DNA, 3.5 kb HBV RNA, and total HBV RNA were listed in Supplementary Table S4.

### Statistical analysis

Statistical analyses were performed by SPSS 24.0 (SPSS Inc., Chicago, IL, USA) software. The Student’s *t*-test was used for comparisons of the luciferase activity, mRNA expression, and protein production between the two groups. A comparison of the clinical parameters was performed using the Mann-Whitney test. Categorical variables were expressed as proportions (%, n/n) and analysed by Chi-square/Fisher’s exact test. All *p* values were two-tailed, and *p* < 0.05 was considered statistically significant.

## Results

### C2729T mutation is associated with HBV MTCT

In total, 34 mother-infant pairs with positive maternal HBeAg and high viral loads (>7 log_10_ IU/ml) were enrolled in this study, of whom 17 were in the immunoprophylaxis failure group and 17 were in the immunoprophylaxis success group. As shown in Table 1, no significant differences were found in baseline characteristics between the two groups. Although there was a trend that the number of breast-feeding infants in the immunoprophylaxis success group was more than that in the immunoprophylaxis failure group (*p* = 0.071), we and other groups have proved that the mother-to-child transmission of HBV is irrespective of feeding pattern [18,19]. Therefore, this trend may be due to the relatively small sample size in this study.

**Table 1.** The baseline characteristics between the immunoprophylaxis failure and success group.

	Immunoprophylaxis Success Group	Immunoprophylaxis Failure Group	*p*
**Mothers**			
Number	17	17	
Age (years), median (range)	27 (19–34)	27 (19–34)	1.000
HBsAg (log_10_ IU/ml), median (range)	4.50 (3.97–4.74)	4.54 (3.49–4.88)	0.502
HBeAg (log_10_ IU/ml), median (range)	3.11 (2.32–3.47)	3.19 (2.52–3.48)	0.586
Genotype C2, n (%)	17 (100)	17 (100)	1.000
**Infants**			
Number	17	17	
Gender, male: female	10:7	10:7	1.000
Birth weight (kg), median (range)	3.40 (2.70–4.20)	3.50 (2.60–4.00)	0.317
Parturition manner, vaginal: caesarean	8:9	5:12	0.481
Feeding pattern, breast^a^: artificial	9:8	3:14	0.071

^a^Breast-feeding included mixed feeding.

It has been reported that HBsAg is closely related to HBV MTCT [20]. Therefore, based on the nucleotide mutation rates in full-length HBV genome sequences from mothers in the immunoprophylaxis failure and success groups, we explored the effect of mutations on three HBsAg coding regions (preS1, preS2, and S regions) and two HBsAg promoter regions (SPI and SPII regions) in HBV MTCT. As shown in Table 2, there were no obvious differences in the average nucleotide mutation rates between the two groups in three HBsAg coding regions. In the SPI region, we found that the average nucleotide mutation rate in the immunoprophylaxis success group was significantly higher than that in the immunoprophylaxis failure group (1.55% *vs*. 0.71%, *p* = 0.027), but no significant difference was found in SPII region between the two groups. Furthermore, the average nucleotide mutation rate in the SPI region was the highest among the above five regions. These results revealed that the mutations in the SPI region might make a valuable contribution towards HBV MTCT.

**Table 2.** The average nucleotide mutation rates in the HBsAg-related regions between the immunoprophylaxis failure and success group.

	The average nucleotide mutation rate^a^		*p*
	Immunoprophylaxis Success Group (*n* = 17)	Immunoprophylaxis Failure Group (*n* = 17)	
SPI (2718–2808)	1.55% (24/1547)	0.71% (11/1547)	**0.027**
SPII (2983–3210)	0.80% (31/3876)	0.88% (34/3876)	0.709
preS1 (2848–3204)	0.66% (40/6069)	0.76% (46/6069)	0.516
preS2 (3205–154)	1.39% (39/2805)	1.28% (36/2805)	0.727
S (155–835)	0.34% (39/11577)	0.32% (37/11577)	0.818

^a^The average nucleotide mutation rate = the total nucleotide mutation frequency in the analysed region/(the number of nucleotides in the analysed region × the number of sequences in the group).

**Figure 1.**
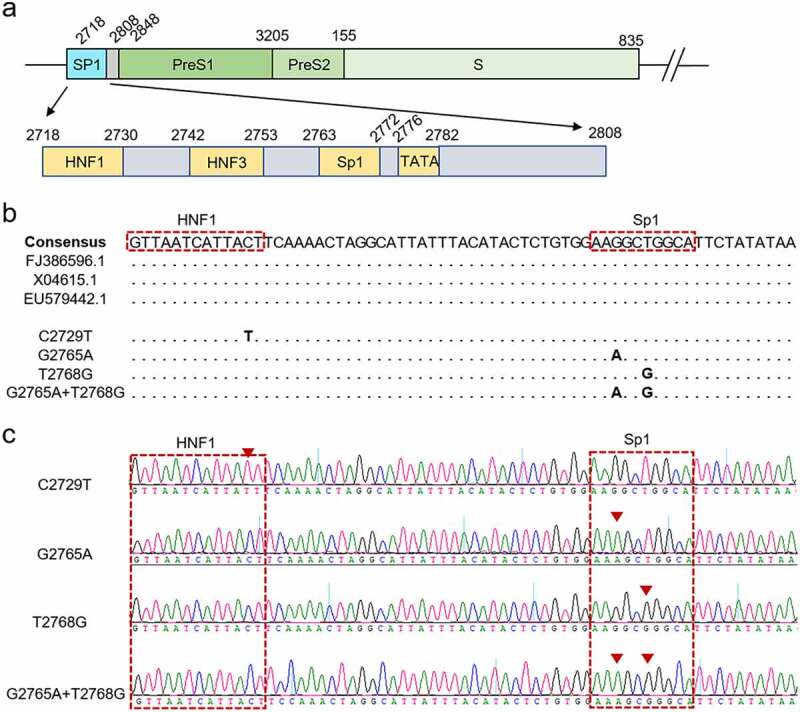
The schematic representation showing the structure of SPI sequence. (a) a schematic diagram showing the structure of SPI. (b) Alignment of SPI nucleotide sequences, including the consensus sequences from Genbank database and the sequences of the mutant HBV strains. (c) the representative peak diagrams of the mutant HBV strains.

As we know, the binding sites of HNF1 (nt 2718–2730), hepatocyte nuclear factor 3 (HNF3) (nt 2742–2753), and Sp1 (nt 2763–2772) are located in the SPI region (Figure 1a). To further investigate the specific mutations of the SPI region associated with HBV MTCT, the nucleotide mutations were further analysed, especially for the binding sites of the three transcription factors and TATA box region. The result revealed that the nucleotide mutation of SPI region was present in 52.94% (9/17) of the mothers in the immunoprophylaxis success group, which was significantly higher than that in the immunoprophylaxis failure group (52.94% *vs*. 5.88%, *p* = 0.007). Among the 9 mothers with nucleotide mutation in the immunoprophylaxis success group, C to T mutation at the 2729th base (C2729T) located at the binding site of HNF1 was present in 44.44% (4/9) of mothers. The other mutations including T to G mutation at the 2768th base (T2768G), G to A mutation at the 2765th base (G2765A), and T2768G+G2765A combined mutation were all located at the binding site of Sp1 and were present in 33.33% (3/9), 11.11% (1/9) and 11.11% (1/9) of mothers, respectively. Since these nucleotide mutations are only present in the immunoprophylaxis success group, indicating that they may contribute to the immunoprophylaxis success of HBV MTCT by affecting the ability of viral production or infectivity.

### C2729T mutation promotes HBsAg release by reducing LHBs expression

**Figure 2.**
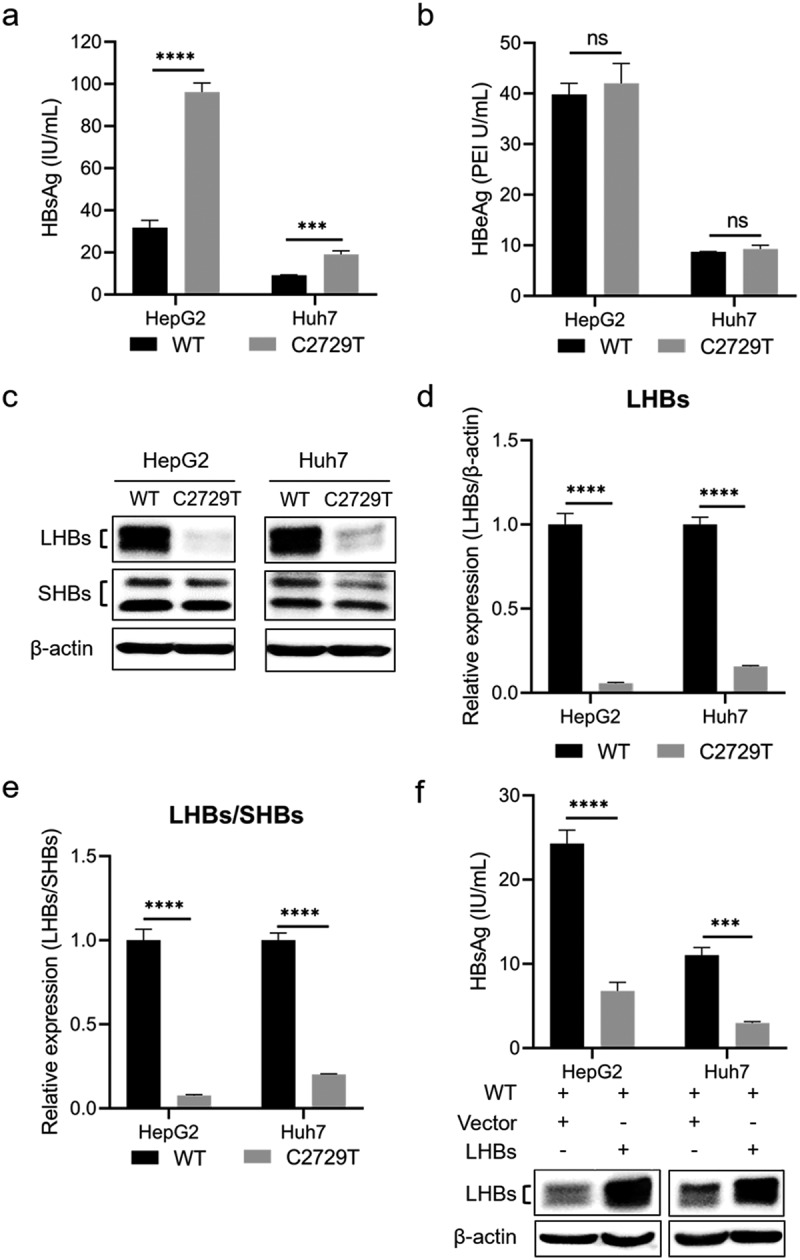
The C2729T mutation promotes HBsAg secretion by reducing LHBs expression. The pBB4.5–HBV 1.2×-WT or pBB4.5–HBV 1.2×-C2729T plasmid and pCDH-Nluc plasmid were co-transfected into HepG2 and Huh7 cells. Cell culture supernatants and cells were harvested at 3 days post-transfection. The levels of (a) HBsAg and (b) HBeAg in the cell culture supernatants were detected by chemiluminescence immunoassays. (c) the levels of intracellular LHBs and SHBs were detected by Western Blot. The β-actin protein was used as the internal control. The levels of LHBs were normalized to (d) the levels of β-actin or (e) the levels of SHBs using ImageJ software. (f) the pBB4.5–HBV 1.2×-WT plasmid, pCDH-LHBs or pCDH vector control plasmid, and pCDH-Nluc plasmid were co-transfected into HepG2 and Huh7 cells, the levels of HBsAg in the cell culture supernatants were detected by chemiluminescence immunoassays, and the levels of intracellular LHBs were detected by Western Blot at 3 days post-transfection. The data were presented as the mean ± SD of three independent experiments and were analysed by Student’s *t*-test. ns-no statistical significance, ***p < 0.001, ****p < 0.0001.

It has been reported that the nucleotide mutation in the binding site of Sp1 can significantly decrease HBV viral load by reducing the transcriptional activity of SPI [15], which supports our speculation that the nucleotide mutations in the SPI region may contribute to immunoprophylaxis success of HBV MTCT. However, the effect of the C2729T mutation on HBV production is not clear yet. To explore the effect of the C2729T mutation on HBV production, the plasmid containing wild-type (pBB4.5-HBV1.2×-WT) or C2729T mutant (pBB4.5-HBV1.2×-C2729T) 1.2 fold HBV genome and nano-luciferase expression plasmid (pCDH-Nluc) used as an internal control plasmid were co-transfected into HepG2 and Huh7 cells. The levels of HBsAg and HBeAg in the cell culture supernatants were detected at 3 days post of transfection. The results showed that the C2729T mutation could significantly increase the level of HBsAg in the cell culture supernatants of both HepG2 and Huh7 cells (Figure 2a), but not affected the level of HBeAg (Figure 2b). Meanwhile, the C2729T mutation significantly decreased the levels of intracellular LHBs but not for SHBs, thus leading to a significantly decreased ratio of LHBs/SHBs (Figure 2c-e). As shown in Supplementary Figure S1a, the transfection efficiency between the transfected cells was comparable, which was assessed by measuring nano-luciferase activity.

For the increased level of HBsAg in the cell culture supernatants, we speculated that the C2729T mutation-mediated reduction of LHBs level might promote HBsAg secretion. To confirm this speculation, pBB4.5-HBV1.2×-WT plasmid, LHBs expression plasmid (pCDH-LHBs), or vector control plasmid (pCDH), and pCDH-Nluc plasmid were co-transfected into HepG2 and Huh7 cells, then the level of HBsAg in the cell culture supernatants was detected at 3 days post of transfection. The results showed that overexpressing LHBs could significantly decrease the level of HBsAg in the cell culture supernatants of both HepG2 and Huh7 cells (Figure 2f and Supplementary Figure S1b), which suggested that LHBs could suppress SHBs release, and conversely, the C2729T mutation-mediated reduction of LHBs level could promote HBsAg release.

### C2729T mutation suppresses HBV production

**Figure 3.**
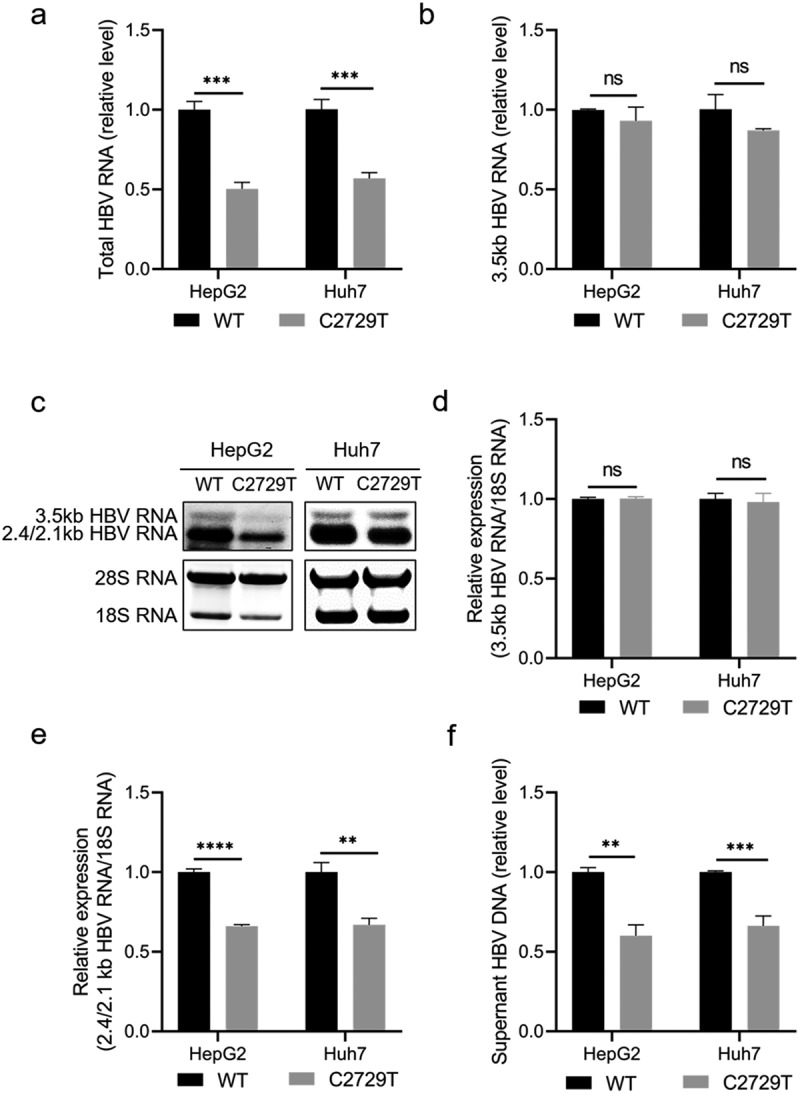
The C2729T mutation suppresses HBV production. The pBB4.5-HBV1.2×-WT or pBB4.5-HBV1.2×-C2729T plasmid and pCDH-Nluc plasmid were co-transfected into HepG2 and Huh7 cells. The cells and cell culture supernatants were harvested at 3 and 5 days post-transfection to detect the levels of HBV RNA and DNA, respectively. The levels of intracellular total HBV RNA (a) and 3.5 kb HBV RNA (b) were detected by RT-qPCR. The *ACTB* mRNA was used as the internal control. (c) the levels of intracellular HBV mRNAs including 3.5 kb and 2.4/2.1 kb HBV RNAs were detected by Northern Blot. The 28S and 18S rRNAs were used as the internal control. The levels of (d) 3.5 kb and (e) 2.4/2.1 kb HBV RNAs were normalized to the levels 18S RNA using ImageJ software. (f) the levels of HBV DNA in the cell culture supernatants were detected by qPCR. The data were presented as the mean ± SD of three independent experiments and were analysed by Student’s *t*-test. ns-no statistical significance, **p < 0.01, ***p < 0.001, ****p < 0.0001.

To further investigate the effect of C2729T mutation in HBV production, its effects on the levels of HBV RNA and HBV DNA were detected. RT-qPCR results showed that the C2729T mutation could significantly reduce the level of total HBV RNA in both HepG2 and Huh7 cells (Figure 3a), but not for 3.5 kb HBV RNA (Figure 3b). In line with this, Northern blot further demonstrated that the C2729T mutation could significantly reduce the level of 2.4/2.1 kb HBV RNA, but not affected the level of 3.5 kb HBV RNA (Figure 3c-e).

It has been reported that the reduced LHBs level or reduced ratio of LHBs/SHBs can inhibit virion secretion [6]. Consistently, the C2729T mutation could indeed significantly reduce the level of HBV DNA in the cell culture supernatants of both HepG2 and Huh7 cells (Figure 3f). These results suggested that the C2729T mutation could inhibit HBV secretion and thus suppress HBV production.

### LHBs can rescue the reduced HBV production mediated by C2729T mutation

**Figure 4.**
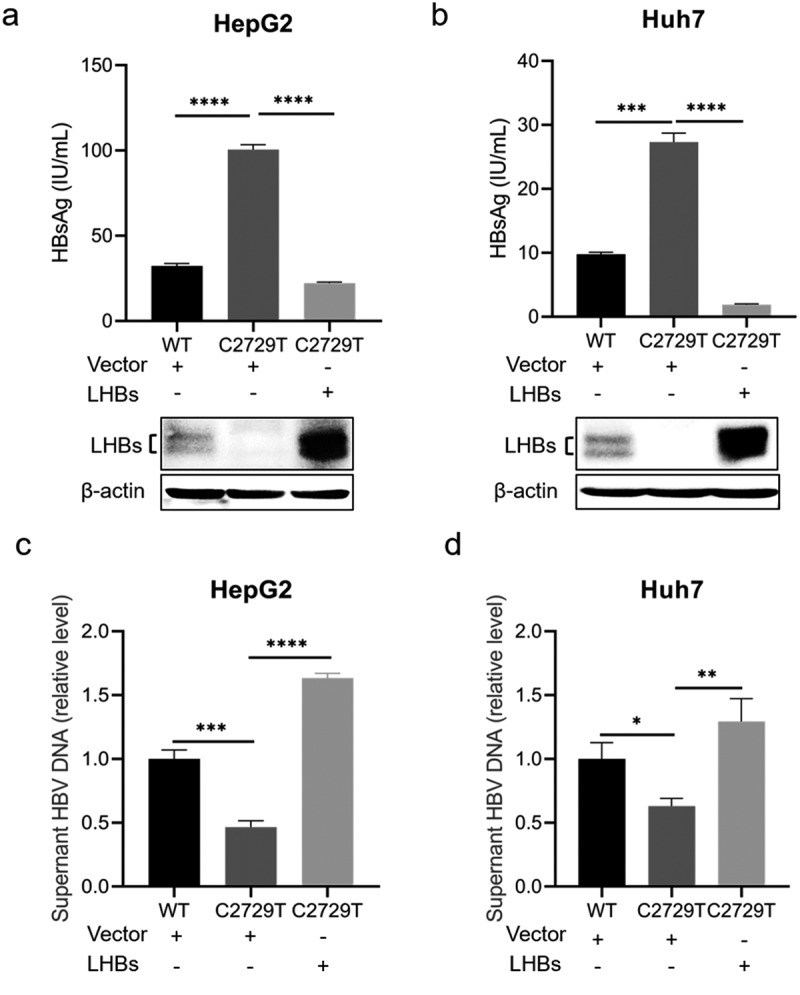
LHBs can rescue the reduced HBV production mediated by the C2729T mutation. The pBB4.5-HBV1.2×-WT or pBB4.5-HBV1.2×-C2729T plasmid, pCDH-LHBs or pCDH vector control plasmid, and pCDH-Nluc plasmid were co-transfected into HepG2 and Huh7 cells. Cell culture supernatants and cells were harvested at 3 and 5 days post-transfection to detect the levels of HBsAg and HBV DNA, respectively. (a & b) the levels of HBsAg in the cell culture supernatants were detected by chemiluminescence immunoassays, and the levels of intracellular LHBs were detected by Western Blot in HepG2 and Huh7 cells. The β-actin protein was used as the internal control. (c & d) the levels of supernatant HBV DNA were detected by qPCR in HepG2 and Huh7 cells. The data were presented as the mean ± SD of three independent experiments and were analysed by Student’s *t*-test. ns- no statistical significance, *p < 0.05, **p < 0.01, ***p < 0.001, ****p < 0.0001.

To further explore whether the reduced HBV production was mediated by the decreased LHBs, pBB4.5-HBV1.2×-WT or pBB4.5-HBV1.2×-C2729T plasmid, pCDH-LHBs plasmid or pCDH vector control plasmid, and pCDH-Nluc plasmid were co-transfected into HepG2 and Huh7 cells. The levels of HBsAg, HBeAg, and HBV DNA in the cell culture supernatants were detected. The results showed that the C2729T mutation-mediated increase of HBsAg level in the cell culture supernatants was rescued by overexpressing LHBs (Figure 4a and b, and Supplementary Figure S1c, d). Meanwhile, the C2729T mutation-mediated decrease of HBV DNA level in the cell culture supernatants was also rescued by overexpressing LHBs (Figure 4c and d). However, the level of HBeAg was not affected by both the C2729T mutation and LHBs overexpression (Supplementary Figure S2). The above results further confirmed that LHBs could suppress SHBs release but promote HBV production, indicating that LHBs played an important role in the C2729T mutation-mediated aberrant HBV secretion.

### C2729T mutation downregulates the transcriptional activity of SPI by attenuating the binding ability of HNF1 to SPI

**Figure 5.**
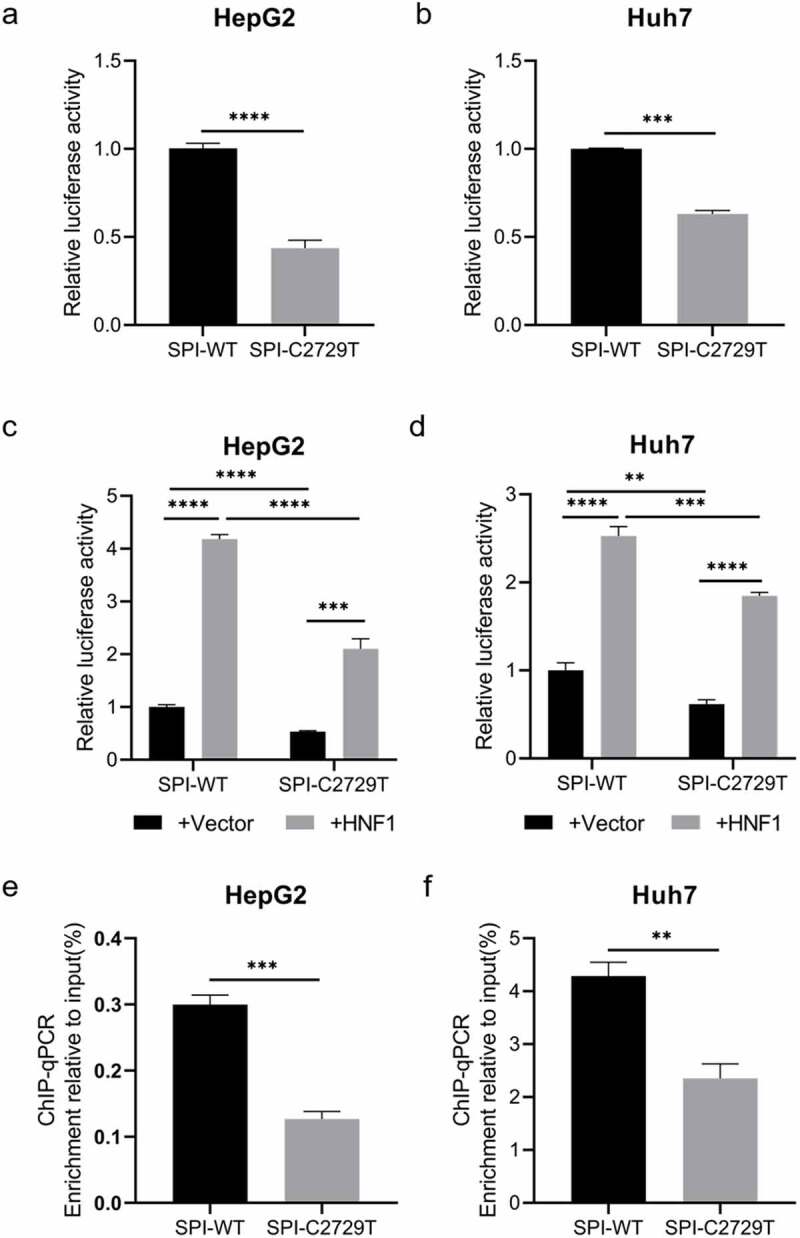
The effect of the C2729T mutation on the transcriptional activity of SPI. The transcriptional activities of SPI were detected by dual-luciferase reporter assays at 48 h after (a & b) co-transfection of pGL3-SPI or pGL3-SPI-C2729T plasmid and pRL-TK plasmid or (c & d) co-transfection of pGL3-SPI or pGL3-SPI-C2729T plasmid, pcDNA3.1-HNF1-HA or pcDNA3.1 vector control plasmid, and pRL-TK plasmid in HepG2 and Huh7 cells. (e & f) the binding activities between HNF1 and HBV SPI were detected by ChIP-qPCR at 48 h after co-transfection of pBB4.5-HBV1.2×-WT or pBB4.5-HBV1.2×-C2729T plasmid and pCDNA3.1-HNF1-HA in HepG2 and Huh7 cells. The data were presented as the mean ± SD of three independent experiments and were analysed by Student’s *t*-test. **p < 0.01, ***p < 0.001, ****p < 0.0001.

As shown in Figure 1, the C2729T mutation altered the binding site of HNF1 in the SPI region, therefore the dual-luciferase reporter assay was performed to determine whether the transcriptional activity of SPI was affected by the C2729T mutation. The wild-type SPI luciferase reporter plasmid (pGL3-SPI) or the C2729T mutant SPI luciferase reporter plasmid (pGL3-SPI-C2729T), and renilla luciferase expression plasmid (pRL-TK) were co-transfected into HepG2 and Huh7 cells, and then the transcriptional activity of SPI was detected at 2 days post of transfection. The results showed that the C2729T mutation could significantly downregulate the transcriptional activity of SPI in both HepG2 and Huh7 cells (Figure 5a and b). Further, pGL3-SPI or pGL3-SPI-C2729T plasmid, HNF1 expression plasmid (pcDNA3.1-HNF1-HA) or vector control plasmid (pcDNA3.1), and pRL-TK plasmid were co-transfected into HepG2 and Huh7 cells, and then the transcriptional activity of SPI was detected. The results revealed that ectopic HNF1 could enhance the transcriptional activities of both the wild-type and C2729T mutant SPI, whereas the ability of ectopic HNF1 to enhance the transcriptional activity of the C2729T mutant SPI was significantly weaker than that of the wild-type SPI (Figure 5c and d).

Furthermore, ChIP-qPCR was performed to explore the effect of C2729T mutation on the binding ability of HNF1 to SPI. As shown in Figure 5e and f, the enrichment of the C2729T mutant SPI sequence pulled down by HNF1 was much less than that of the wild-type SPI sequence. Therefore, the C2729T mutation-attenuated binding ability of HNF1 to SPI contributed to the downregulated transcriptional activity of HBV SPI.

## Discussion

In this study, we found that the average nucleotide mutation rate of the HBV SPI region in the immunoprophylaxis success group was significantly higher than that in the immunoprophylaxis failure group, and a substitution (C2729T) in the binding site of HNF1 make a major proportion among the nucleotide mutations in SPI region, which suggested C2729T mutation might be associated with HBV MTCT.

Further, we found that the C2729T mutation significantly decreased the level of intracellular LHBs, but the level of HBsAg in the cell culture supernatants significantly increased, which indicated that the C2729T mutation promoted the secretion of non-infectious subviral particles (SVP). As the most abundant envelope protein, SHBs can be efficiently released as the main component of SVP. It has been reported that SHBs and LHBs are co-carboxy termini, and thus they can form heteropolymers whose phenotype depends on the relative amount of surface proteins, that is, relatively low amounts of LHBs lead to the secretion of SHBs, while relatively high amounts of LHBs lead to the retention of SHBs [21,22]. Later reports confirmed that the release of SHBs can be inhibited by LHBs in a dose-dependent manner due to the formation of heteropolymers, and a too-high LHBs/SHBs ratio will lead to intracellular retention of all forms of HBsAg [6,8,9]. Consistently, we found that the C2729T mutation-mediated decrease of LHBs expression could lead to the decrease of LHBs/SHBs ratio, and subsequently promote the secretion of HBsAg or HBV SVP. In terms of HBV production, the C2729T mutation could significantly decrease the levels of HBV DNA in the cell culture supernatants, which was rescued by overexpressing the ectopic LHBs. It has been reported that two matrix domains (MD1 and MD2) of Golgi-processed HBsAg in the membrane of multivesicular body (MVB) contact with HBV nucleocapsids, and subsequently drive the inward budding process [23]. The inward-budded nucleocapsids are sorted into the endosomal sorting complex required for transport (ESCRT) complexes and subsequently release outside the hepatocytes [24]. Since MD1 is located at LHBs, and MD2 is located at SHBs, indicating that both LHBs and SHBs are necessary for the release of HBV particles [25–28]. Therefore, the C2729 mutation-induced reduction of LHBs protein might lead to the accumulation of HBV in the hepatocytes, and thus lead to the reduction of HBV production.

HNF1 is a key transcription factor binding to nt 2718–2730 in the HBV SPI region and is necessary for initiating the transcription of 2.4 kb HBV RNA [29–31]. Considering the C2729T mutation altered the binding site of HNF1 in the SPI region, the influence of this mutation on the binding ability of HNF1 to SPI was explored in this study. We found that the transcriptional activity of SPI was significantly reduced by the C2729T mutation with or without HNF1, even though HNF1 could enhance the transcriptional activity of both the wild-type and C2729T mutant SPI. Meanwhile, the ability of HNF1 to enhance the transcriptional activity of the C2729T mutant SPI was weaker than that of the wild-type SPI. Further, we found that the C2729T mutation could attenuate the binding ability of HNF1 to SPI, which might contribute to the C2729T mutation-mediated downregulation of the SPI transcriptional activity.

As we know, the levels of HBsAg, HBeAg, and HBV DNA were positively correlated with each other, and the maternal HBsAg and HBeAg could be used as surrogate markers of HBV DNA and able to predict HBV MTCT [32–36]. However, the high HBsAg levels but low HBV viral loads are present in a small proportion of the patients chronically infected with HBV. This study revealed that the LHBs expression of the C2729T mutant HBV was much lower than that of the wild-type HBV, and thus could lead to the higher HBsAg levels but the lower HBV viral loads, which could partially explain the above clinical phenomenon. However, there are several limitations in this study. First, the potential effect of the C2729T mutation in HBV MTCT was only explored *in vitro* due to the lack of animal models for HBV MTCT. Second, this study was performed with a relatively small number of mothers with chronic HBV infection. The limiting factors of the number were the strict inclusion criteria and the insufficient serum. Since genotype C2 was the main genotype in this cohort, we only enrolled the mother-infant pairs infected with genotype C2 HBV to reduce the influence from the heterogeneity of different genotypes. In addition, a large amount of serum was used to detect HBV serological and virological indicators; thus, the remaining serum was insufficient to extract HBV DNA to amplify the full-length HBV genomes.

In summary, this study first finds that the nucleotide mutations in the SPI region of HBV genome may be associated with the immunoprophylaxis effect of HBV MTCT. The C2729T mutation in the SPI region can downregulate the transcriptional activity of SPI by attenuating the binding ability of HNF1 to SPI, and subsequently reduce HBV production by suppressing LHBs expression, which indicates that the C2729T mutation may contribute to immunoprophylaxis success of HBV MTCT. Taken together, this study supplements the virological factors potentially affecting the immunoprophylaxis effect of HBV MTCT and may be helpful to formulate an optimal immunization strategy for the infants born to the mothers chronically infected with HBV.

## Supplementary Material

Supplemental Material

## Data Availability

The authors confirm that the data reported in this study are available within the article and/or its supplementary materials.
