# Supplementary material for: C2729T mutation associated with HBV mother-to-child transmission reduces HBV production via suppressing LHBs expression
Source: Virulence. 2023 Mar 15;14(1):2189676. doi: 10.1080/21505594.2023.2189676 (PMC10026911; doi:10.1080/21505594.2023.2189676)
Supplement: Supplemental Material [file KVIR_A_2189676_SM3377.docx]

**C2729T mutation associated with HBV mother-to-child transmission reduces HBV production via suppressing LHBs expression**

**Authors:** Minmin Liu^a,#^, Yarong Song^a,b,#^, Yi Li^a^, Xingwen Yang^a,b^, Hui Zhuang^a^, Jie Li^a,*^, and Jie Wang^a,b,*^

**Table S1**. The primers used for HBV genome amplification

| Primer names | Primers sequences (5’-3’) |
| --- | --- |
| P1-F | TGAACATGCAGTTAATCATTATTTCAAAACTAGGCATTATTTAC |
| P2-R | GTAAATAATGCCTAGTTTTGAAATAATGATTAACTGCATGTTCA |

F: forward, R: reverse.

**Table S2**. The primers used for HBV direct sequencing

| Primer names | Primers sequences (5’-3’) | Location |
| --- | --- | --- |
| RC2 | CTCCRAAAGASACCAAATA | 2270-2252 |
| LF1 | TTTTTCACCTCTGCCTAATCA | 1821-1841 |
| LF4 | GTCACCATATTCTTGGGAAC | 2816-2835 |
| LF6s | TGTCCTCCAATTTGTCCTGG | 347-366 |
| H5 | GTGCCATTTGTTCAGTGGTTCG | 683-704 |
| BF2 | GCGGGTCGTCCTTTGTTTAC | 1409-1428 |
| LR1 | AAAAAGTTGCATGGTGCTGG | 1825-1806 |
| H1 | CGAGAAAGTGAAAGCCTGCT | 1101-1082 |
| PR3 | CATAGCAGCAGGATGAAGAGGA | 423-402 |
| LR4s | TTGAGGTCCCAATCTGGATT | 2984-2965 |
| LR7 | GGGTTCAAATGTATACCCAA | 839-820 |
| LF7 | TATTGGGGGCCAAGTCTGTA | 752-771 |
| H3 | CTGTTGTTAGACGACGAGGCA | 2340-2360 |

**Table S3**. Sequences of the primers used for plasmid construction

| Primer names | Primers sequences (5’-3’) |
| --- | --- |
| Site-directed mutation F | TGAACATGCAGTTAATCATTATTTCAAAACTAGGCATTATTTAC |
| Site-directed mutation R | GTAAATAATGCCTAGTTTTGAAATAATGATTAACTGCATGTTCA |
| pCDH-LHBs-F | GTTTTGACCTCCATAGAAGATTCTAATGGGAGGTTGGTCTTCCAAAC |
| pCDH-LHBs-R | CCGCAATTTAAATGAATTCTTCGAAGGATCCTCAAATGTATACCCAAAGACAAAAG |
| pCDNA3.1-HNF1-HA-F | GAAGGATCCGCCGCCACCATGGTTTCTAAACTGAGC |
| pCDNA3.1-HNF1-HA-R | CTTTCTAGATTAAGCGTAATCTGGAACATCGTATGGGTAGAATTCCTGGGAGGAAGAGGCCAT |

F: forward, R: reverse.

**Table S4**. Sequences of the primers used for HBV DNA and HBV RNA quantification

| Quantitative markers | Primers sequences（5’-3’） |
| --- | --- |
| HBV DNA | F: CCGTCTGTGCCTTCTCATCTG |
|  | R: AGTCCAAGAGTCCTCTTATGTAAGACCTT |
| 3.5kb HBV RNA | F: GAGTGTGGATTCGCACTCC |
|  | R: GAGGCGAGGGAGTTCTTCT |
| Total HBV RNA | F: AAGCCACCCAAGGCACAG |
|  | R: GCACCAGCACCATGCAAC |
| *ACTB* RNA | F: ACTGTGCCCATCTACGAGG |
|  | R: CAGGCAGCTCGTAGCTCTT |

F: forward, R: reverse.

**
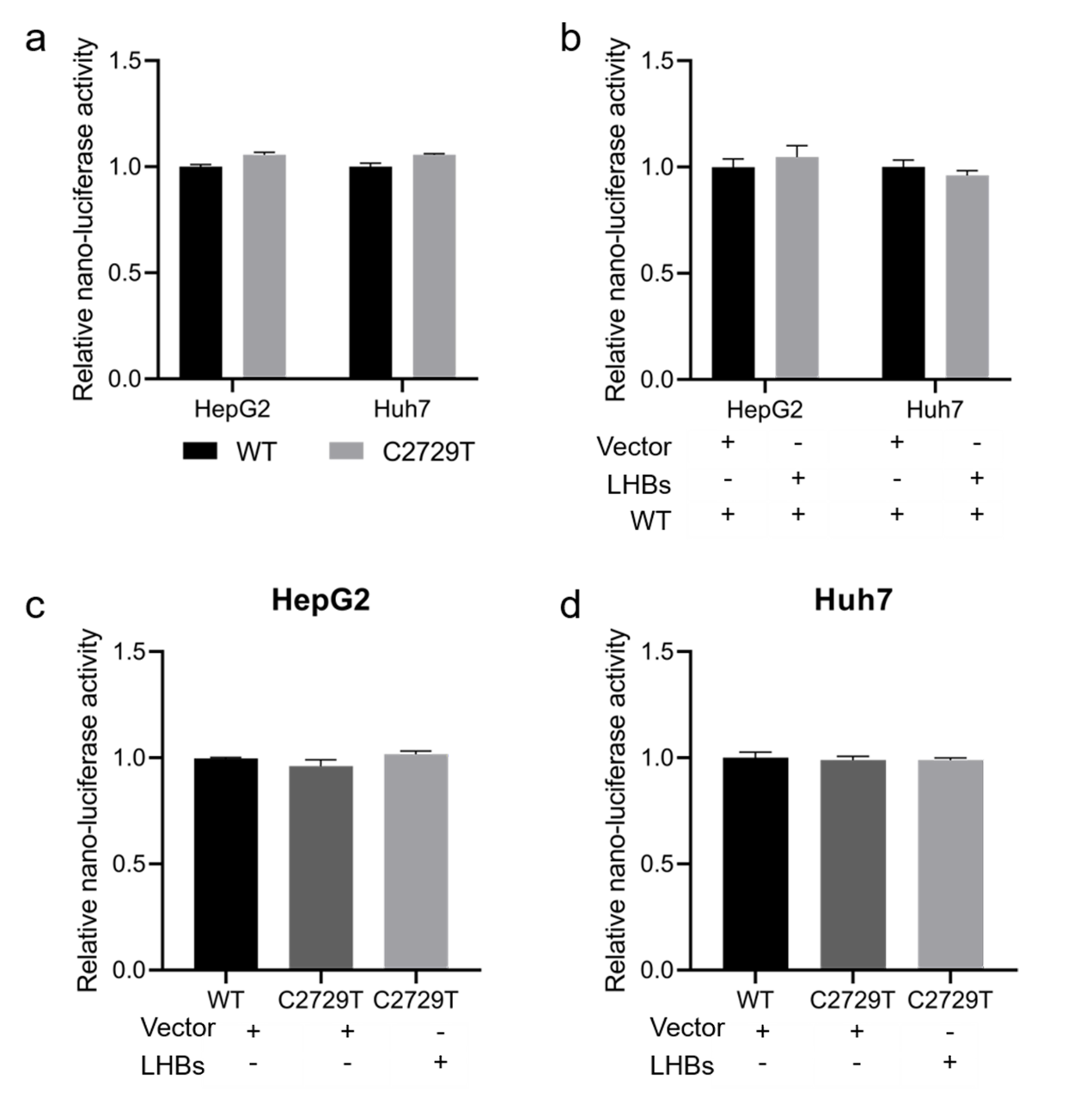
**

**Figure S1.** The transfection efficiencies are assessed by detecting Nano-luciferase activities. (a) The pBB4.5-1.2×-WT or pBB4.5-1.2×-C2729T plasmid and nano-luciferase expression plasmid (pCDH-Nluc) were co-transfected into HepG2 and Huh7 cells. At 72 h post-transfection, the Nano-luciferase activities were detected by Nano-luciferase assays. (b) The pBB4.5-1.2×-WT plasmid, pCDH-LHBs or pCDH vector control plasmid, and pCDH-Nluc plasmid were co-transfected into the HepG2 and Huh7 cells. At 72 h post-transfection, the Nano-luciferase activities were detected by Nano-luciferase assays. The pBB4.5-HBV1.2×-WT or pBB4.5-HBV1.2×-C2729T plasmid, pCDH-LHBs or pCDH vector control plasmid, and pCDH-Nluc plasmid were co-transfected into (c) HepG2 and (d) Huh7 cells. At 72 h post-transfection, the Nano-luciferase activities were detected by Nano-luciferase assays.

**
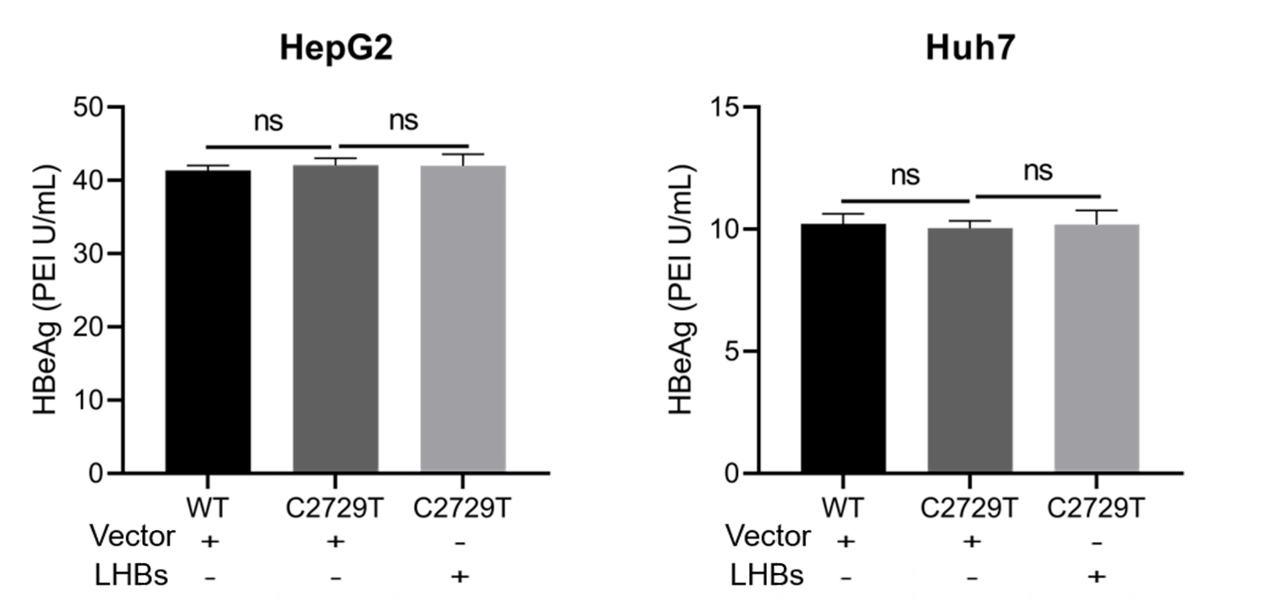
**

**Figure S2.** The effect of ectopic LHBs in the HBeAg expression of the C2729T mutant HBV. The pBB4.5-HBV1.2×-WT or pBB4.5-HBV1.2×-C2729T plasmid, pCDH-LHBs or pCDH vector control plasmid, and pCDH-Nluc plasmid were co-transfected into HepG2 and Huh7 cells. The levels of HBeAg in the cell culture supernatants were detected by chemiluminescence immunoassays at 3 days post-transfection. The data were presented as the mean ± SD of three independent experiments and were analyzed by Student’s *t*-test. ns- no statistical significance.
